# Understanding Dementia Carer Experiences Before Admission to a Residential Aged Care Facility: Implications for Integrated Care

**DOI:** 10.1177/07334648241261454

**Published:** 2024-07-18

**Authors:** Jennifer White, Dane Falcioni, Roslyn Barker, Julie Bajic-Smith, Chitra Krishnan, Elise Mansfield, Carolyn Hullick

**Affiliations:** 1 School of Medicine and Public Health, 5982 The University of Newcastle, Callaghan, NSW, Australia; 2 454568 Hunter Medical Research Institute, New Lambton Heights, NSW, Australia; 3 5260 Hunter New England Local Health District, New Lambton Heights, NSW, Australia; 4 Wise Care, Malabar, NSW, Australia; 5 Australian Commission of Safety and Quality in Health Care, Sydney, NSW, Australia

**Keywords:** access, carer, decision-making, dementia, qualitative

## Abstract

In-depth understanding of dementia carer experience can assist clinicians by providing insight into dementia onset, symptoms and management, and help conceptualize and understand the pattern of dementia progress over time and what help is needed. We undertook a qualitative study to understand dementia carers experiences of providing care and reasons for admission to a residential aged care facility (RACF). Three themes were identified: (1) Challenges in the path to diagnosis and care, leading to delays accessing support; (2) Carer role impacted by living circumstances; and (3) Variation in decision support prior to admission to a RACF. Identifying dementia carer experiences, reinforces the need for more timely diagnosis, referral for support and interventions to promote better quality of life for a people living with dementia and their carer and to delay premature RACF placement.

**What this paper adds**

• The importance of early diagnosis, assessment, and referral to services that support carers and people living with dementia (PLWD) to remain at home supported remains critical.
• Primary care and hospital clinicians are uniquely placed to identify care needs of both PLWD and their carers including future care planning.
• Gaps in timely access to assessment and support remain the complexity of navigating the aged care system and long wait times for assessment and community support, Home Care Packages, contribute to carer burden, and increase the likelihood of earlier admission to RACF.

**Applications of study findings**

• Early intervention is essential, and we highlight the need for greater initiatives to assist clinicians in the identification and management of people with cognitive decline.
• There is a need to better prepare carers for their role and provide support across their carer journey in order to help them manage symptoms in the PLWD, respond to arising issues and delay admission to a RACF. Closely aligned is support for their own mental health.
• There is a need to simplify the process for access to diagnosis and health and support services for PLWD as their health declines including accessing home care packages and respite.

## Introduction

In Australia, two thirds of people who receive community aged care services transitioned to a residential aged care facility (RACF) (Australian Bureau of Statistics, 2018). Of the approximately 371,000 Australians accessing RACFs, over half included people living with dementia (PLWD) (Australian Institute of Health and Welfare, 2023). In 2020, 54% of PLWD lived in RACFs and 46% lived in the community with support of primary carers of whom 90% provided continuous rather than episodic care, and over half were carers of partner (Australian Bureau of Statistics, 2020). This is consistent internationally where family carers are considered to be an essential taskforce in caring for people (Bell et al., 2019).

Internationally, countries have established dementia care guidelines addressing a range of key priorities from emphasis on early diagnosis and management to high-quality end-of-life care (Nakanishi et al., 2015). How well guidelines are implemented can influence how long the PLWD remains at home. In Australia, models of care rely on primary care clinicians, most commonly general practitioners (GPs) to diagnose and provide ongoing care for older patients, including those with dementia (Herzog et al., 2015). In the context of a busy primary care setting, it may be difficult to diagnose dementia and its management can be complex (Pimlott et al., 2009). Known barriers to optimal dementia management in primary care include lack of training and confidence among clinicians, lack of time during consultations, difficulty managing the PLWD who struggles to adhere to dementia management plans, and lack of support for carers and recognition of carer tress (Mansfield et al., 2019). Results of a scoping review of dementia carers support needs, highlighted a lack of person-centered care approach (PCC) which considers the carers perspective (Holt Clemmensen et al., 2021). PCC is routinely emphasized in dementia and aged care policy and practice (Dyer et al., 2016; Fazio et al., 2018) and refers to a philosophy of care that centers on meeting individual needs, through interpersonal engagement, and collaborative care partnerships between the person with dementia, their carers, and treating clinicians (Brooker, 2003). PCC interventions, whereby the person, carer, and clinicians work in a partnership to develop a care plan (Røsvik et al., 2013), have systematically been shown to reduce agitation, neuropsychiatric symptoms and depression, and improve the quality of life of PLWD (Kim & Park, 2017).

Against this backdrop is evidence of high rates of burden in carers of PLWD including poor quality of life and poor physical and mental health linked to the need to provide assistance with activities of daily living (ADLs), long hours of care, emotional stress, social isolation, and financial hardship (Brodaty & Donkin, 2009; Tatangelo et al., 2018; Thompson et al., 2007). More recent evidence highlights the complexities of providing care during the COVID-19 (COVID) pandemic (Ng et al., 2020). To complete this picture, there is increasing evidence of challenges in accessing and navigating the aged care service system, leading to unmet needs (Xie et al., 2023) not being met by health professionals (Stirling et al., 2010). In Australia, the federal government subsidizes the aged care system based on the older person’s care needs as well as their ability to pay. A standardized Aged Care Assessment Team (ACAT) assesses older people for community support programs and institutional care. Based on this assessment, subsidized home care packages (HCPs) are offered at four levels. However, wait times mean many PLWD are assigned lower level packages than needed, or must wait months for packages to become available (Hill, 2022).

As dementia progresses the burden of care often exceeds the resources of the informal carer leading to a decision towards admission to a RACF (Pimlott et al., 2009). For example PLWD experience multiple challenges including changed cognition and behavior, ADL deficits, poor mobility, incontinence, depression, and higher number of hospitalizations (Afram et al., 2014; Sverdrup et al., 2018; Young et al., 2020). Past evidence exploring the dementia carer experiences highlights the need for pre-planning, (ongoing) support, and reassurance (Mansfield et al., 2023). Concern stems from evidence that the current health and aged care system is fragmented and difficult to navigate for older people with and without dementia, many of whom lack digital and health literacy (Sanfey & Hastie, 2000) and carers of PLWD report the need for help using the My Aged Care online portal to access assessment and support (Mansfield et al., 2023).

Promoting person-centered and integrated care in the context of dementia remains difficult. While there is no widely accepted definition of integrated care (Goodwin, 2016), it is commonly referred to as an approach to overcome health care fragmentation through the provision of seamless coordination of care services across a range of settings to ensure delivery of holistic and collaborative care focused on the individual needs of the PLWD and their carer (Shaw et al., 2011). As part of a broader research program exploring how to better support carers of PLWD, we sought to further our understanding of the reasons carers could no longer care for the PLWD at home and the associated experience of access to diagnosis, community support, respite, and transition care. Exploring dementia care giver experiences, as well as how support is accessed, will help inform local service delivery.

## Methodology

An interpretive qualitative approach was deemed appropriate to address the research aims. Carers were eligible for inclusion if they were listed as the primary carer of a PLWD or person with cognitive impairment who had been admitted to a RACF in the Hunter region in the past 24 months or were inpatients awaiting RACF placement from a metropolitan hospital; being unable to be discharged home due to safety concerns for the PLWD and their carer. Potential participants were identified and invited to participate by the RACF or hospital staff, they were provided with detailed study information and had the opportunity to ask questions about the research. All participants provided informed consent. Recruitment occurred between Feb and June 2023. This qualitative study employed the use of semi-structured interviews and was informed by the Consolidated criteria for reporting qualitative research (COREQ) checklist (Tong et al., 2007). Approval for this project was obtained from the Hunter New England Health Human Research Ethics Committee (Ref: 2022/ETH02560).

## Data Collection

Semi-structured interviews (*n* = 25), ranging from 30–60 minutes, were conducted by a skilled qualitative interviewer (JW) at a mutually convenient time and location. Interviews were guided by an interview schedule. Interviews began by asking participants to share their experience of being a carer, including an exploration of services carers had accessed prior to admission of the person living with dementia. Questions probed their experience of integrated care towards service access, future planning or not. Identified themes informed continuing data collection and sampling continued until thematic saturation (two co-coders agreeing that no new themes were emerging) was achieved.

## Data Analysis

Interviews were audio-recorded, transcribed verbatim, and checked for transcription errors. Two authors (JW and DF), one a female occupational therapist with extensive clinical experience working with people living with dementia and their carers, and the other a male psychology student and research assistant, independently coded data using an inductive thematic approach (Braun & Clarke, 2012). Analysis followed a three-phase approach involving (i) identifying units of meaning by reading the transcripts line-by-line, (ii) grouping units into categories to assist with data retrieval, irrespective of the research question, and (iii) examining relationships between codes to form themes. Consistency of findings was upheld through regular discussion of interpretations between the research team to confirm codes and theme development. Consistency of our data was upheld using several strategies, including immersion in data; reflexive analysis, and peer debriefing (Krefting, 1991).

## Results

The majority of carers were adult children (*n* = 19) and six were wives. Carers were living in both metropolitan (*n* = 20) and regional settings (*n* = 5). Eleven carers were living with the PLWD. Fourteen PLWD were diagnosed after symptoms became problematic, four were diagnosed by a geriatrician; however, seven were never formally diagnosed.

Three themes were identified towards the challenges experiences by carers of PLWD and the reasons they could no longer care for the PLWD at home thus precipitating the need for admission to a RACF.

1. Challenges in the path to diagnosis and care, leading to delays accessing support
2. Carer role impacted by living circumstances
3. Variations in decision support prior to admission to a RACF

## Challenges in the Path to Diagnosis and Care Lead to Delays Accessing Support

This theme underscored the multifaceted challenges participants faced in navigating the diagnosis and care of PLWD, emphasizing the importance of timely and comprehensive support structures. The diverse experiences shared by participants shed light on the need for improved communication, accessibility, and understanding within the health care system to better assist those affected by dementia and their caregivers.

### Access to Diagnosis and Care

Participant reports suggested that initial access to dementia diagnosis and referral to support was ad hoc and some PLWD never received a diagnosis of dementia until admission to a RACF. Participants reported challenges in obtaining timely dementia diagnoses and support, with symptoms often being attributed to external stressors such as work, being a carer, or the death of spouse.

He had been doing that job for the last 20 years and was quite highly regarded in the company. And then he was falling behind just and getting inundated, forgetting things. (P23, wife)

It is not that we didn’t believe dad … we just thought that maybe mum was just horrible to him because she was sick of doing things for him. (P13, daughter)

Participants typically contacted a general practitioner (GP) in the first instance when symptoms were becoming problematic. However, there was variation in the experience of interactions with GPs, with some participants reporting difficulties in receiving effective diagnosis and support. Changing GPs was a strategy employed by some participants to access more comprehensive support and referrals. For example, one participant reported the PLWD was misdiagnosed as having “depression for about two years” (P25, wife) which caused significant distress and confusion until they changed GPs.

I said, “I need help with mum and dad” and his [GP] answer to me was, “What do you want me to do?”. Well, I didn’t know what needed to be done. I didn’t know what avenues to go down. Whereas after I changed GPs for them [parents], that next GP wrote me all sorts of referrals and things like that - to get someone to come and assess them. (P24, daughter)

The involvement of geriatricians in diagnosis and care planning process also varied. Some participants reported that their geriatrician primarily focused on updating the severity of the dementia diagnosis, while others found reassurance in the acknowledgment of their caregiving role.

I just did not feel like that he was too helpful but then as I left, he said to me, “You are doing a good job.” Nobody had ever said that to me, that was really good to hear. (P17, daughter)

### Navigating the Aged Care System

For many participants the onset of symptoms was nebulous, and support was only sought when the care giving situation was already strained, and often following a hospital admission.

The hospital organised for the [home] care package. (P2, wife)

Most participants struggled to negotiate the My Aged Care online processes to obtain an ACAT assessment and HCP support. For several participants a lack of awareness of the My Aged Care system led to not applying for services or delays in accessing help until they were struggling to manage escalating symptoms.

I just didn’t know what I could get or what I needed, so I didn’t know, so I never did. I just did everything myself, that’s what I did, I just did everything. (P20, wife)

No, we were in the process of doing that [applying] and then she had a fall in the lounge room. (P16, son)

Further COVID added complexities to an already challenging situation, causing delays in appointments, assessments, and communication issues which exacerbated participants’ emotional strain.

By the time the appointment was due, which was supposed to be six months. It turned out 18 months due to COVID - and we were not allowed to ring them or email them. So we couldn’t ring and say, ”Look she is escalated, can we have an assessment.” They said that is not the way we do it, we are too busy and that you have to wait for your turn to come up. (P13, daughter)

In response to feelings of distress and confusion, participants reported they relied on friends and family members for assistance in navigating the system—highlighting the need for external support in understanding and instigating available services.

When the lady [assessor] came out I didn’t have a clue what she was saying, so I had a friend help me to do it… She [friend] was my voice, which was very helpful, and she got the ball rolling for me. (P8, wife)

Participants who were children reported they were more able to access My Aged Care website due to their computer literacy but often still reported significant challenges towards understanding and navigating the process. It was considered a “complicated” (P11, son) system that was not integrated with other key government portals such as My Gov (government services online) and Centrelink (social security payments and services). Many participants reflected on the need for support such as contact person or “liaison support” (P5, daughter) and better communication and advice.

I have a degree; I am not an idiot. I cannot figure out all these processes and the communication with them has been dreadful. (P5, daughter)

Overall, lengthy waits for services were common following an ACAT assessment. Many participants reported they did not receive a HCP, or the recommended level of HCP, prior to the PLWD being admitted to a RACF. This was compounded when the PLWD “refused” (P19, daughter) home care services. Despite repeated attempts to negotiate with the PLWD, a few participants did not receive relief from their caring demands.

We tried to get a bit of home care, like meals a bit of companionship but mum refused all of it because she had me. She wanted me to do it – even though it was basically to give me a break. (P17, daughter)

Very few participants reported they were referred to Dementia Australia (a not-for-profit service providing information, education, and services) for support and only a few participants undertook self-education and “read a lot about it.” (P13, daughter) highlighting potential gaps in accessing available resources for information and support.

I give myself credit that I did a lot of research and I really think that I have done everything possible that there is out there for him. (P23, wife)

## Carer Role Impacted by Living Circumstances

This theme portrays the challenges participants faced in managing complex living scenarios. From navigating family dynamics to accessing professional help and addressing legal aspects, participants journey was marked by difficulties that often led to hospital admission. Where available, access to supportive professionals was pivotal in guiding participants through these intricate situations.

### Managing Deteriorating Symptoms

All participants reported they cared for a PLWD for several years and that care demands increased due to deterioration in symptoms. Providing dementia related care was intensified by the presence of other chronic conditions (cardiovascular disease, chronic obstructive pulmonary disease, and kidney disease), depression, and difficulty engaging with the environment due to hearing impairment. Participants reported that as the PLWD function deteriorated, there was an increase in the need to monitor and provide support with ADLs such as showering and changing into clean clothes. Similarly, more assistance was required for instrumental ADLs (IADLs) such as meal preparation, home maintenance and navigating the environment. Due to the extent of daily demands two participants moved in with their parent and three participants had their parent move in with them as were insufficient to meet all the needs of the PWLD.

Dad could prepare basic meals, but he would leave the stove on. (P16, father)

We would come home from work, and she would be sitting in the dark with the fan going - because the fan switch was right next to the light switch, and she just couldn’t tell the difference. (P7, daughter)

Participants recounted challenges related to safety concerns, especially concerning wandering behaviors in the PLWD. Participants worry was exacerbated when the PLWD was lost, and they were trying to find them—if they had left the home unknowingly. Alternatively, participants felt they “had to put whatever I want to do on hold” (P6, son) for fear they would wander.

I was sitting by the front door all day, terrified and with the door locked - just trying to keep him inside. (P25, wife)

Sleep disruption was a common experience and impacted quality of life, especially in carers who were spouses. The ability to manage fatigue was reportedly essential for sustaining effective care and “to function” (P2, wife).

I was very tired. If he had a sleep of an afternoon, I would sit on the lounge not intending to but I would be out like a light….. and by about 6 o’clock I very ready for bed (P18, wife)

Balancing caregiver responsibilities with other commitments also reportedly became more intricate. Participants, particularly those “maintaining work” (P4, daughter) or caring for additional family members, found it challenging to navigate multiple roles.

And then the next thing my sister was terminally ill, and it all just kind of fell apart … what with mum being sick and dad’s dementia. (P21, daughter)

Over time, participants expressed concern that the PWLD ceased participation in pleasurable activities and had become increasing sedentary which was manifested in non-purposeful behavior such as “staying in bed” (P15, son) or “sitting” (P16, daughter). Subsequently participants struggled to help the PLWD engage with their environment and reported that the symptoms of dementia meant “there was just nothing that he could do” (P2, wife) such as read, or watch television. This emotional impact underscored the multifaceted challenges faced by participants as they witnessed the progressive decline in the quality of life of the PLWD.

### Managing Symptoms of Changed Behaviors

An additional demand for many participants was managing symptoms of changing behaviors. Within months or over several years the extent of changed behaviors created an additional and significant burden for some participants ability to assist with ADLs and IDLs. These participants experienced regular verbal and physical aggression from the PLWD.

He would get violent towards me, and I would end up with bruises on my arms and … he didn’t actually hit me, but he was going to a few times. (P20, wife)

Daily caregiving became a series of struggles as the PLWD exhibited changed behaviors, ranging from inappropriate dressing, wanting to keep driving, and resistance towards taking medication.

He would go outside on hot days in a tracksuit, you know it is hot and he is in a tracksuit. He didn’t know how dress for the weather. I would say to him, “You need to come inside and put shorts on and a T-shirt.” I would get a reply back, “I know what I am doing, leave me alone.” (P8, wife)

Not showering, not eating, argumentative about medication ….. I would hand them to him and he would either throw them at me or throw them on the floor or saying, “You are trying to poison me.” (P24, daughter)

External factors such as the COVID lockdown introduced new layers of difficulty. For one participant COVID lockdowns created profound confusion in her mother who missed seeing her grandchildren and thought they were being intentionally being withheld from her resulting in “nasty texts” and threats. (P13, daughter).

For some participants the cumulation of providing years of care pushed them to “breaking point” (P17, daughter). This was associated with feelings of significant stress, and some struggled to manage their emotions and maintain empathy for the PLWD.

I hated what he was sometimes. (P2, wife)

Few participants tried to access respite however due to a lack of availability of respite or the inability of a RACF to manage changed behaviors, participants didn’t get the break they craved. Most participant didn’t persevere when the first attempt failed.

I think that he might have lasted that night [in respite], but then the next night they rang me and asked if I could come and get him…He was disturbing the other residents, so that didn’t work really. (P2, wife)

### Managing Complex Living Scenarios

Some participants relayed the experience of distress trying to care for a PLWD when a home situation led to difficulties facilitating care and negotiating with family members—to the extent that issues of neglect were of concern. The challenges associated with dealing with a complex home situation made it difficult for participants to respond to care needs in an ideal timeframe and access help from health professionals. This was exacerbated for participants who were located a considerable distance from the PLWD or when the PLWD was being supported at home by another parent who was struggling to manage the deteriorating situation.

If mum was not well enough to cook nobody ate. (P3, daughter)

It was overwhelming for dad. He was ringing my brother up more than myself and just sort of you know not coping which was understandable. (P5, daughter)

Participant’s reports suggested they felt powerless when they observed a deteriorating home scenario where the PLWD refused to attend to ADLs, wandered, or was verbally and physically aggression towards a parent.

Mum would get very, very agitated and smash things if he was asleep. Every now and then she would just lean over and punch him and wake him up and yeah so, she was hitting a lot. (P5, daughter)

One participant who was estranged from her brother (who had moved in with her mother). This participant described difficulty having access to her mother to also provide support. This was more difficult during COVID.

He [brother] wouldn’t let me help, it was a very difficult relationship. He just wasn’t giving my mother the correct care. And then COVID happened, and I couldn’t get down to my mother. He cut the phone off, so I had no way of communicating with my mother. (P9, daughter)

All participants, where possible neglect was a factor, reported difficulty accessing HCPs to help alleviate the situation due to or service delays or rural proximity. Similarly lack of knowledge regarding legal aspects, such as obtaining guardianship and power of attorney, contributed to delays initiating services. As such, attempts to escalate concerning situations to aged care services and the PLWD’s GP proved difficult.

They wouldn’t accept that how bad things were, and it was very difficult to deal with My Aged Care. I tried desperately…. I thought that GP could go to the home, I asked him could he do a home visit. He wouldn’t do that. (P9, daughter)

Some participants expressed gratitude for supportive healthcare professionals, particularly GPs, who played a crucial role in facilitating care, promoting family communication, and aiding in future planning. Access to professionals helped participants navigate their role as carer, validating their experiences and fostering an awareness of the necessity for institutional care.

The GP was very communicative, so my dad eventually gave permission for her to talk to me. She would ring me up after the appointments just to give me an update, worked with me in terms of talking dad through putting mum into care - as that is you know where she needs to go. (P5, daughter)

Hospitalization occurred when the situation was dire, due to aggression and neglect of personal hygiene, ultimately culminating in the transition to a RACF.

And when I found her, the house was a mess. My mother’s clothes were absolutely filthy. She was putting the same clothes back on every day. She wasn’t showering, her scalp. She had cradle cap all through her hair, it was disgusting. I was so heart broken. (P9, daughter)

So, in hospital they had to medicate her because she was extremely aggressive, hitting or pulling her clothes off herself…. Her hair - we ended up having to cut it all off because she had not had it brushed in God knows how long. (P5, daughter)

## Variations in Decision Support Prior to Admission to a RACF

The decision-making process leading to RACF admission was complex and influenced by factors such as immediate care demands, safety concerns, and recommendations from healthcare professionals. The involvement of children and their expressed concerns becomes a crucial element in participants considering and accepting the transition to institutional care.

### Future Planning

Participants reported limited engagement in decision-making processes prior to admission to a RACF for the PLWD. Future planning was often postponed, especially when participants felt they were managing care at home and therefore didn’t consider the need for institutional care. Participants focus generally, was on immediate, hands-on care rather than long-term planning. Indeed, few participants “discussed” (P19, daughter) admission to a RACF with the PLWD as function deteriorated, and hence, no formal plans were made for residential care. Participants often struggle alone with their decision-making due particularly concerning whether to seek emergency help or alternative care options. The following narrative reflects the internal conflicts participants faced in determining the best course of action for the PLWD wellbeing.

It was really hard; it was a struggle in the aspect of … am I doing the right thing? Or should they be in a care facility? (P24, daughter)

Despite the reported benefit of access to HCPs, day centers, private nurses and respite towards the provision of day to day practical and emotional support, there was limited reported discussion with healthcare professionals towards future planning. Only few participants reported having a close relationship with their own GP or the PLWD GP to navigate future planning.

She [GP] just said we need to get her [PLWD] into the hospital you know get this sorted out - she probably really needs to go into care because I can see you are at the end of your tether. (P1, daughter)

Participant reports also suggested that there was variation in the involvement of the PLWD in diagnosis and care planning, both within discussions among family and health professionals. In hindsight participants reported that early, integrated care may have helped them navigate the care journey including preparing the PLWD for the benefit of services.

His GP didn’t really ever say, “You have got dementia”. They might have indicated it to me, but not to him. I thought was a big mistake - but he wasn’t given that opportunity [to be involved in decisions]. (P2, wife)

### Hospital Instigated

All participants reported that the PLWD started to experience increasing hospital admissions in the months leading up to admission to a RACF as dementia progressed. Managing falls, incontinence, dehydration and comorbidities were made more complex in the context of deteriorating symptoms. Many participant reports suggested they struggled to manage these scenarios without help and that it was often a hospital admission when support was facilitated, including the instigation of conversations suggesting the need of community services or admission to a RACF.

He was weeing himself and then he had lots of aches and pains and falling. (P8, wife)

So, like for the tipping point… possibly the fall. (P16, daughter)

The recommendation for RACF admission was often suggested to participants due to concerns for the safety of both the participants and the PLWD. Safety issues, such as falls and changed behaviors, became tipping points leading to the realization that care at home was no longer safe. Participants reported that an external perspective, usually from hospital healthcare professionals, was the prompt for discussions about the future.

They were telling me that I could not bring him home because it was not safe for me to have him at home - for me or for him. (P23, wife)

There was variability in how involved participants felt about their involvement in decision-making process for RACF admission. The emotional impact of these decisions varied, with some participants expressing a pragmatic acceptance, while others find it alarming and difficult to accept.

Yeah, in a way they were pretty horrible decisions but, in a way, they were made for me. (P25, wife)

I was quite surprised, though I realised she could not go home. I just could not really think this was likely to happen. (P4, daughter)

While many participants, especially spouses tried to manage at home for as long as possible, the management of increasing falls and incontinence and subsequent hospitalizations was difficult. These participants reported that their children expressed concerned for the participant’s health which often precipitated discussion about the need for admission to a RACF.

The kids said, “Mum, I think we best talk about this, you can’t do it. You tried …” (P18, wife)

However, participants who were spouses found the adjustment most difficult, “I mean, 50 years and all of a sudden now we are separated. It is horrible.” (P8, wife). Indeed, some spouses reflected that they felt like being, “A widow now.” (P20, wife)

Ongoing distress was so profound for one participant who considered suicide as a means of gaining relief from the grief and distress when her husband (PLWD]) of 60 years was not adjusting to the RACF and continually wanted to return home.

Sometimes when I am driving, I think about driving into a tree and ending it for both of us. (P2, wife)

## Discussion

This study provides detailed insight into the experience of carers of PLWD in providing care and reasons for making a decision towards admission to a RACF, despite advancement in dementia care in recent years. By identifying gaps in coordinated dementia care we highlight care areas that can be better tailored to the needs of PLWD and their carer. A concerning finding in this study was the extent to which carer struggles where exacerbated by difficulty accessing the aged care service system, including practical support and help for increasing dementia symptoms and changed behaviors. Results echo a recent survey examining the prevalence and type of unmet needs experienced in carers of PLWD (Mansfield et al., 2023) including support for emotional wellbeing, accessing health and aged care services, accessing appropriate dementia support services, and information needs.

Our findings reinforce the imperative in obtaining timely diagnosis of dementia and early referral for a community ACAT assessment in identifying the support needed to assist PLWD to remain living at home, including referral to multi-disciplinary and social community support programs. Consistent with previous research we identified that future planning was difficult and seldom initiated (Tilburgs et al., 2018)—being typically facilitated following an acute hospital admission. This reinforces the need for comprehensive Geriatric assessments, including access to geriatricians, to ensure accurate diagnosis, and the prudent provision of education, referral for support, liaison with GPs, and advanced care planning to support carers (Clevenger et al., 2012; Kable et al., 2019).

It is predicted that the number of Australians with dementia will more than double by 2058 (Australian Bureau of Statistics, 2020). As a result, improving the recognition and management of dementia in the community is increasingly important. Concern stems from findings in our study that delays in accessing care for diagnosis, treatment, planning and assistance led to a crisis where institutional care was the only option. Consistent with previous research we identified that carers of PLWD were often referred to ACAT only after experiencing prolonged periods of stress (Bruce et al., 2002) and therefore could not manage with further delays in accessing community HCPs. Even when an ACAT assessment was conducted, participants experienced significant delays in receiving approved care packages. As a result, we advocate for enhanced community and clinician education and process that ensure early referral to ACAT in order to commence the process, rather than waiting for a crisis.

We highlight the extent of carer stress managing complex symptoms and changed behavior as dementia progressed, reinforcing the need for increased support as well as education to prevent premature entry to a RACF. As a starting point, we posit the need for increased uptake of the Australian government funded health assessments for people over 75 years (75+HA) in primary care, including a focus on cognition as well as carer stress. The 75+HA is available annually and designed to identify health issues and conditions that are preventable or amenable to early intervention with the goal of improving the health of older adults. In this context, consideration is also needs to be given towards future planning, including facilitating substitute decision makers and legal guardians as people living with dementia continue to deteriorate and may not be able to make decisions for themselves. However our past research highlight that even when referrals were made following a 75+HA, there was a need for these recommendations and referrals to be actively followed so that PLWD did not fall through the cracks (White et al., 2024). As such there are implications for Australia’s universal health insurance scheme to promote integrated care including ensuring referrals are completed.

The strength of this study lies in the exploration of a heterogenous sample of carers of people living with dementia. We acknowledge the potential for reporting bias and that responding participants may have had different experiences to non-responders including access to primary care where differing models of care exist. We also acknowledge the lack of representation from cultural and linguistically diverse carers and involvement of PLWD towards their experiences of integrated care.

### Conclusion

There remain gaps in effective integrated care for PLWD and their carers. Our study highlights that primary care and hospital clinicians are uniquely placed to identify care needs of both PLWD and their carers including future care planning. The importance of early diagnosis, assessment and referral to services that support carers and PLWD to stay at home remains critical. However, gaps in timely access to assessment and support remain the complexity of navigating the aged care system and long wait times for assessment and HCPs contributes to carer burden, increasing the likelihood of earlier admission to RACF. Our results also highlight the importance of ongoing education of both health care professionals and carers towards the recognition and management of dementia in the health and community settings.

## Data Availability

Available on request.

## References

[bibr1-07334648241261454] Afram B. Stephan A. Verbeek H. Bleijlevens M. H. Suhonen R. Sutcliffe C. Raamat K. Cabrera E. Soto M. E. Hallberg I. R. Meyer G. Hamers J. P. H. (2014). Reasons for institutionalization of people with dementia: Informal caregiver reports from 8 European countries. Journal of the American Medical Directors Association, 15(2), 108–116. 10.1016/j.jamda.2013.09.012 24238605

[bibr2-07334648241261454] Australian Bureau of Statistics . (2020). Dementia in Australia. Available from: https://www.abs.gov.au/articles/dementia-australia (Accessed 9 November 2023).

[bibr3-07334648241261454] Australian Bureau of Statistics . (2018). Disability, ageing and carers, Australia: Summary of findings. Available from: https://www.Abs.Gov.Au/statistics/health/disability/disability-ageing-and-carers-australia-summary-findings/latest-release (Accessed 1 December 2023).

[bibr4-07334648241261454] Australian Institute of Health and Welfare . (2023). People using aged care. Available from: https://www.Gen-agedcaredata.Gov.Au/topics/people-using-aged-care (Accessed 9 November 2023).

[bibr5-07334648241261454] Bell J. F. Whitney R. L. Young H. M. (2019). Family caregiving in serious illness in the United States: Recommendations to support an invisible workforce. Journal of the American Geriatrics Society, 67(S2), S451–S456. 10.1111/jgs.15820 31074854

[bibr6-07334648241261454] Braun V. Clarke V. (2012). Thematic analysis. American Psychological Association.

[bibr7-07334648241261454] Brodaty H. Donkin M. (2009). Family caregivers of people with dementia. Dialogues in Clinical Neuroscience, 11(2), 217–228. 10.31887/DCNS.2009.11.2/hbrodaty 19585957 PMC3181916

[bibr8-07334648241261454] Brooker D. (2003). What is person-centred care in dementia? Reviews in Clinical Gerontology, 13(3), 215–222. 10.1017/s095925980400108x

[bibr9-07334648241261454] Bruce D. G. Paley G. A. Underwood P. J. Roberts D. Steed D. (2002). Communication problems between dementia carers and general practitioners: Effect on access to community support services. Medical Journal of Australia, 177(4), 186–188. 10.5694/j.1326-5377.2002.tb04729.x 12175321

[bibr10-07334648241261454] Clevenger C. K. Chu T. A. Yang Z. Hepburn K. W. (2012). Clinical care of persons with dementia in the emergency department: A review of the literature and agenda for research. Journal of the American Geriatrics Society, 60(9), 1742–1748. 10.1111/j.1532-5415.2012.04108.x 22985144

[bibr11-07334648241261454] Dyer S. M. Laver K. Pond C. D. Cumming R. G. Whitehead C. Crotty M. (2016). Clinical practice guidelines and principles of care for people with dementia in Australia. Australian Family Physician, 45(12), 884–889. 27903038

[bibr12-07334648241261454] Fazio S. Pace D. Maslow K. Zimmerman S. Kallmyer B. (2018). Alzheimer’s Association dementia care practice recommendations. Oxford University Press US. 10.1093/geront/gnx182 29361074

[bibr13-07334648241261454] Goodwin N. (2016). Understanding integrated care. International Journal of Integrated Care, 16(4), 6. 10.5334/ijic.2530 PMC5354214 28316546

[bibr14-07334648241261454] Herzog A. Gaertner B. Scheidt-Nave C. Holzhausen M. (2015). ‘We can do only what we have the means for’general practitioners’ views of primary care for older people with complex health problems. BMC Family Practice, 16, 1–11. 10.1186/s12875-015-0249-2 25886960 PMC4371843

[bibr15-07334648241261454] Hill T. (2022). Understanding unmet aged care need and care inequalities among older Australians. Ageing and Society, 42(11), 2665–2694. 10.1017/s0144686x21000222

[bibr16-07334648241261454] Holt Clemmensen T. Hein Lauridsen H. Andersen‐Ranberg K. Kaae Kristensen H. (2021). Informal carers’ support needs when caring for a person with dementia–a scoping literature review. Scandinavian Journal of Caring Sciences, 35(3), 685–700. 10.1111/scs.12898 32781496

[bibr17-07334648241261454] Kable A. Pond D. Hullick C. Chenoweth L. Duggan A. Attia J. Oldmeadow C. (2019). An evaluation of discharge documentation for people with dementia discharged home from hospital–A cross-sectional pilot study. Dementia, 18(5), 1764–1776. 10.1177/1471301217728845 28875738

[bibr18-07334648241261454] Kim S. K. Park M. (2017). Effectiveness of person-centered care on people with dementia: A systematic review and meta-analysis. Clinical Interventions in Aging, 12, 381–397. 10.2147/CIA.S117637 28255234 PMC5322939

[bibr19-07334648241261454] Krefting L. (1991). Rigor in qualitative research: The assessment of trustworthiness. American Journal of Occupational Therapy, 45(3), 214–222. 10.5014/ajot.45.3.214 2031523

[bibr20-07334648241261454] Mansfield E. Cameron E. C. Boyes A. W. Carey M. L. Nair B. Hall A. E. Sanson-Fisher R. W. (2023). Prevalence and type of unmet needs experienced by carers of people living with dementia. Aging & Mental Health, 27(5), 904–910. 10.1080/13607863.2022.2053833 35356837

[bibr21-07334648241261454] Mansfield E. Noble N. Sanson-Fisher R. Mazza D. Bryant J. (2019). Primary care physicians’ perceived barriers to optimal dementia care: A systematic review. The Gerontologist, 59(6), e697–e708. 10.1093/geront/gny067 29939234

[bibr22-07334648241261454] Nakanishi M. Nakashima T. Shindo Y. Miyamoto Y. Gove D. Radbruch L. Van Der Steen J. T. (2015). An evaluation of palliative care contents in national dementia strategies in reference to the European Association for Palliative Care white paper. International Psychogeriatrics, 27(9), 1551–1561. 10.1017/S1041610215000150 25678323

[bibr23-07334648241261454] Ng R. Lane N. Tanuseputro P. Mojaverian N. Talarico R. Wodchis W. P. Bronskill S. E. Hsu A. T. (2020). Increasing complexity of new nursing home residents in Ontario, Canada: A serial cross-sectional study. Journal of the American Geriatrics Society, 68(6), 1293–1300. 10.1111/jgs.16394 32119121

[bibr24-07334648241261454] Pimlott N. J. Persaud M. Drummond N. Cohen C. A. Silvius J. L. Seigel K. Hollingworth G. R. Dalziel W. B. (2009). Family physicians and dementia in Canada: Part 2. Understanding the challenges of dementia care. Canadian Family Physician, 55(5), 508–509. 10.1017/S0959259813000014 19439708 PMC2682312

[bibr25-07334648241261454] Røsvik J. Brooker D. Mjorud M. Kirkevold Ø. (2013). What is person-centred care in dementia? Clinical reviews into practice: The development of the VIPS practice model. Reviews in Clinical Gerontology, 23(2), 155–163. 10.1017/S095925981300001

[bibr26-07334648241261454] Sanfey A. G. Hastie R. (2000). Judgment and decision making across the adult life span: A tutorial review of psychological research. Psychology Press.

[bibr27-07334648241261454] Shaw S. Rosen R. Rumbold B. (2011) What is integrated care (Vol. 7, pp. 1–23). Nuffield Trust.

[bibr28-07334648241261454] Stirling C. Andrews S. Croft T. Vickers J. Turner P. Robinson A. (2010). Measuring dementia carers' unmet need for services-an exploratory mixed method study. BMC Health Services Research, 10(1), 1–10. 10.1186/1472-6963-10-122 20465782 PMC2875230

[bibr29-07334648241261454] Sverdrup K. Bergh S. Selbæk G. Røen I. Kirkevold Ø. Tangen G. G. (2018). Mobility and cognition at admission to the nursing home–a cross-sectional study. BMC Geriatrics, 18(1), 1–8. 10.1186/s12877-018-0724-4 29378518 PMC5789666

[bibr30-07334648241261454] Tatangelo G. McCabe M. Macleod A. You E. (2018). “I just don’t focus on my needs.” the unmet health needs of partner and offspring caregivers of people with dementia: A qualitative study. International Journal of Nursing Studies, 77, 8–14. 10.1016/j.ijnurstu.2017.09.011 28982034

[bibr31-07334648241261454] Thompson C. A. Spilsbury K. Hall J. Birks Y. Barnes C. Adamson J. (2007). Systematic review of information and support interventions for caregivers of people with dementia. BMC Geriatrics, 7, 1–12. 10.1186/1471-2318-7-18 17662119 PMC1951962

[bibr32-07334648241261454] Tilburgs B. Vernooij-Dassen M. Koopmans R. van Gennip H. Engels Y. Perry M. (2018). Barriers and facilitators for GPs in dementia advance care planning: A systematic integrative review. PLoS One, 13(6), Article e0198535. 10.1371/journal.pone.0198535 29924837 PMC6010277

[bibr33-07334648241261454] Tong A. Sainsbury P. Craig J. (2007). Consolidated criteria for reporting qualitative research (COREQ): A 32-item checklist for interviews and focus groups. International Journal for Quality in Health Care, 19(6), 349–357. 10.1093/intqhc/mzm042 17872937

[bibr34-07334648241261454] White J. Norton G. Pond D. Khaing K. Dolja-Gore Byles J. Carey M. (2024). General practitioner and practice nurses perspectives on implementation of the 75+ health assessment: Implications for clinical practice. Journal of Advanced Nursing Manuscript. in press. 10.1111/jan.16354 PMC11810496 39073187

[bibr35-07334648241261454] Xie Y. Hamilton M. Peisah C. Anstey K. J. Sinclair C. (2023). Navigating community-based aged care services from the consumer perspective: A scoping review. The Gerontologist, 64(2), gnad017. 10.1093/geront/gnad017 37120292

[bibr36-07334648241261454] Young Y. Papenkov M. Hsu W.-H. Shahid F. Kuo Y.-H. (2020). Permanent transition of homecare recipients with dementia to nursing homes in New York State: Risk factors. Geriatric Nursing, 41(5), 553–558. 10.1016/j.gerinurse.2020.02.006 32216955

